# High-risk human papillomavirus genotyping in cervical cancers in Tanzania

**DOI:** 10.1186/s13027-024-00596-1

**Published:** 2024-08-05

**Authors:** Gad Murenzi, Edda Vuhahula, Asteria Kimambo, Subira Matiku, Obed Tuyishime, Edwin Liwa, Thomas Habanabakize, Eulade Rugengamanzi, Atuganile Malango, Gallican Kubwimana, Kathryn Anastos, Philip E. Castle

**Affiliations:** 1 Research for Development, Kigali, Rwanda; 2 Muhimbili University of Health and Allied Sciences, Dar Es Salaam, Tanzania; 3 Kairuki University, Dar es Salaam, Tanzania; 4 Catholic University of Health and Allied Sciences, Mwanza, Tanzania; 5 Arusha Lutheran Medical Centre, Arusha, Tanzania; 6 King Faisal Hospital, Kigali, Rwanda; 7 Butaro Cancer Center of Excellence, Burera, Rwanda; 8 Muhimbili National Hospital, Dar Es Salaam, Tanzania; 9 Albert Einstein College of Medicine, Bronx, NY USA; 10 National Cancer Institute, National Institutes of Health, Bethesda, MD USA

**Keywords:** HPV, HIV, Women living with HIV, Cervical cancer, Tanzania

## Abstract

**Background:**

High-risk human papillomavirus (hrHPV) infection causes almost all cervical cancer. Women living with human immunodeficiency virus (Women living with HIV: WLWHIV) are at a six-fold increased risk of developing cervical cancer. This study assessed hrHPV types in cervical cancer by HIV status and histologic subtypes at Muhimbili National Hospital (MNH) in Tanzania.

**Methods:**

This cross-sectional study used formalin-fixed paraffin-embedded (FFPE) archived tissue blocks of cervical carcinomas diagnosed in the Department of Anatomical Pathology at MNH from January to December 2020. Tissue sections were tested for 15 HPV genotypes (16, 18, 31, 33, 35, 39, 45, 51, 52, 53, 56, 58, 59, 66, and 68) using the Ampfire assay. The distribution of HPV genotypes was assessed and compared by HIV status and histologic subtypes.

**Results:**

The mean age ± standard deviation (*N* = 227, with valid HPV results) was 55 ± 12.9 years, 28.6% (*n* = 65) were WLWHIV, and squamous cell carcinoma (SCC) was the most common histologic subtype (91.2%). Most cervical carcinomas (81.1%, *n* = 184) tested positive for hrHPV with HPV16 (44.1%), HPV18 (15.9%), HPV35 (8.4%) and HPV45 (5.7%) being the most common HPV types. hrHPV was higher among older women with 64.5%, 85.1% and 81.3% among 30–40, 41–60 and ≥ 61-year-old women, respectively (*p* = 0.033). HPV16 was more commonly detected in SCC (47.8%) than in adenocarcinomas (5%) (*p* < 0.0001). There was no difference in hrHPV positivity by HIV status.

**Conclusions:**

We found a high proportion of hrHPV among cervical carcinomas diagnosed in Tanzania. Rolling out HPV vaccines that target more hrHPV types than HPV16/18, especially HPV35 and HPV45, could optimize protection against cervical cancer in Tanzania.

**Supplementary Information:**

The online version contains supplementary material available at 10.1186/s13027-024-00596-1.

## Background

High-risk human papillomavirus (hrHPV) infection, the most common sexually transmitted infection [1], causes almost all carcinomas of the uterine cervix [2]. Carcinoma of the uterine cervix (cervical cancer) is the fourth most common and the fourth leading cause of cancer death among women globally, with 660,000 new cases and 350,000 deaths reported in 2022 [3].

The 2022 GLOBOCAN estimates of the global cancer burden place cervical cancer as the first or second cause of cancer morbidity and mortality among women in most countries in sub-Saharan Africa (SSA) [3], which has the highest burden of human immunodeficiency virus (HIV) infection globally [4]. Cervical cancer age-standardized incidence rates in SSA are estimated to be ten times greater than those in high-income countries such as New Zealand, Australia and Western European countries [3]. Cervical cancer is the leading cause of cancer morbidity and mortality in Tanzania, with 10,241 (25.3% of all cancers) new cases and 6,525 (24.2%) deaths reported in 2020 [5].

Women living with HIV (WLWHIV) are at increased risk of cervical pre-cancer and cancer compared to HIV-negative women [6]. Cervical cancer was included as an AIDS-defining malignancy in 1993 by the US Centers for Disease Control and Prevention (CDC) [7]. The 2022 UNAIDS report indicates that the overall HIV prevalence in Tanzania was 4.3% among adults aged 15 to 49 years and 5.6% among women aged 15 to 49 years [8].

There are 12 hrHPV types designated by the World Health Organization’s (WHO) International Agency for Research on Cancer (IARC) as Group I carcinogens in humans including HPV16, 18, 31, 33, 35, 39, 45, 51, 52, 56, 58, and 59 and one, HPV68, is considered a possible carcinogen [9]. Most currently available data on hrHPV in SSA have been derived from hrHPV infection on exfoliated cervical cells from the cervico-vaginal mucosa and not from actual tissue of diagnosed cervical cancers [10–13]. This implies that it is not known which hrHPV types are causing cervical cancer in SSA and Tanzania. We identified only two studies which used cervical cancer tissue, including a study done in Rwanda, which found 96% of cervical cancers to be positive for hrHPV with HPV16 and 18 the most prevalent [14] and another one done in South Africa, which found 88% hrHPV positivity in cervical cancer tissue with HPV16, 18 and 45 being the most common [15].

The distribution of hrHPV types in cervical pre-cancer and cancer diagnosed among WLWHIV compared to HIV-negative women also varies across global regions. A study done in Sweden showed that WLWHIV in SSA were less likely to be covered by the nonavalent HPV vaccine (Gardasil 9) because HPV16 was less prevalent in their cervical intraepithelial neoplasia grade 3 (CIN3) and HPV35 was more prevalent [16].

In addition, a systematic review and meta-analysis showed that HPV35 was more prevalent in Africa than in Asia and that it was more common than HPV18 [17]. This was further studied by Pinheiro et al. who used multiple large US and international epidemiologic studies and found that African American women had more HPV35 and more HPV35-associated precancers compared to other ethnicities [18]. This is concerning because HPV35 is not included in any currently available vaccines including the highly effective nonavalent vaccine. This was also the same finding in a meta-analysis done by Clifford et al., which indicated that HPV45 was more important for cervical cancers in Africa than in other regions [19].

Vaccination against HPV, especially for young girls before they become sexually active, is the primary prevention strategy for cervical cancer recommended globally [20]. HPV vaccines currently available in most low- and middle-income countries (LMIC) including Tanzania only cover HPV16 and 18 [21]. The types of hrHPV that actually cause cervical cancer in SSA and their interaction with HIV infection are not entirely known and currently, evidence suggests that they may be different from other parts of the world [22]. However, it is important to note that studies that include mostly women of African descent show different types with a higher prevalence of HPV35 [18].

Several studies indicate that different hrHPV types are associated with or cause different cervical cancer histologic subtypes. Most cervical cancers are squamous cell carcinomas (SCC), followed by adenocarcinomas (ADC), and then some other rare subtypes such as adeno-squamous carcinoma [23]. Most histologic subtypes, including neuroendocrine carcinomas, are associated with hrHPV infection. HPV16 is more common among SCC and HPV18 is the most common hrHPV type among ADC [24, 25].

In Tanzania, there is a paucity of data on hrHPV types in cervical carcinoma, carcinomas of WLWHIV, and different histologic subtypes. This study aimed to test for hrHPV types in cervical carcinoma tissue and to compare their distribution by HIV status and histologic subtypes. This will provide data on which hrHPV types are causing cervical cancer in this setting, with implications on the appropriateness of the currently available HPV vaccines for the primary prevention of cervical cancer.

## Methods

### Study design, population and setting

This cross-sectional study reviewed archived slides and used formalin-fixed paraffin-embedded (FFPE) tissue blocks from cervical carcinomas for hrHPV genotyping. Our study population was all carcinomas of the uterine cervix diagnosed at the Muhimbili National Hospital (MNH) Central Pathology Laboratory’s (CPL) Department of Anatomical Pathology from January 1st to December 31st, 2020.

### Eligibility criteria

We included participants with confirmed diagnoses of cervical carcinoma, having records of their HIV status, and tissue blocks available in the archive with adequate and viable tissue in the available blocks. Participants with no verifiable HIV status and no available tissue blocks were excluded.

### Data collection methods

#### Data from medical records and archived tissue

The collected data for all variables were entered into an Excel sheet on all the eligible cases identified using a research code as a unique identifier where age in years, parity, HIV status, and diagnosis (histologic subtype) were recorded. HPV testing was then performed on tissue using polymerase chain reaction (PCR) and the hrHPV types present in each tissue specimen were added to the Excel sheet. For histologic diagnosis and subtypes, we also retrieved available slides, and a pathologist in training and a senior pathologist reviewed them blinded to the original diagnosis. A consensus was reached regarding any disagreements. For the missing slides, we made sections from the archived blocks and stained them with hematoxylin and eosin (H&E). Histological subtyping was done according to the 2020 WHO classification of female genital tumors criteria [26].

### HPV testing in tissue

Tissue blocks were retrieved and put on ice before 10–20 μm sections were made using a microtome and placed in a 2 mL tube for transport for HPV testing using the AmpFire assay at the Rwanda Military Hospital research laboratory in Kigali, Rwanda managed by Research for Development under the Einstein-Rwanda Research and Capacity Building Program. The AmpFire HPV genotyping assay (Atila Biosystems Inc., Mountain View, CA, USA) is an isothermal nucleic acid amplification-based, real-time fluorescence detection of 15 HPV genotypes (16, 18, 31, 33, 35, 39, 45, 51, 52, 53, 56, 58, 59, 66, and 68) individually in 4 reaction tubes. Testing was done according to the manufacturer’s protocol [27]. Briefly, an aliquot of the digested tissue was pelleted by centrifugation, the supernatant decanted, and pelleted cells suspended in lysis buffer. The cell suspension was incubated for 90 minutes at 95^o^C to lyse the cells. For each reaction, 2 µL of lysate was mixed with 10 µL of Reaction Mix and 10 µL of one of the four Reaction Mixes. The resulting four reaction tubes for every sample were incubated in the Powergene 9600 fluorescence real-time polymerase chain reaction (PCR) system at 60^o^C with fluorescence from FAM/HEX/ROX/CY5 channels measured every minute.

After running for approximately one hour, the amplification results were interpreted according to exponential curves developed during the process. This experiment run was valid if the negative control showed no exponential curves and the positive control showed exponential curves. The next step was to examine the set of four tubes corresponding to a specimen. Multiplex HPV infections could result in multiple exponential curves for a specimen. If no exponential curve other than internal control (Hex channel in PM-3 tube) is present for a sample, this sample was considered negative. If there was no exponential amplification curve in any of the four tubes or any fluorescence channels, the sample would have failed the test. A failed sample usually indicates that there is not enough DNA in the sample, and it was reprocessed.

Results were classified as positive or negative for any hrHPV, defined here as HPV16, 18, 31, 33, 35, 39, 45, 51, 52, 56, 58, 59, 66, and 68, and for each hrHPV type. To account for multiple hrHPV infections detection, we attributed the cancer to each hrHPV detected and also classified hrHPV test results hierarchically: HPV16 positive, else HPV16 negative but HPV18 or 45 positive, else HPV16, 18, and 45 negative but HPV31, 33, 35, 52, or 58 positive, else negative for HPV16, 18, 31, 33, 35, 52, and 58 but positive for the other hrHPV types. We also classified the hrHPV types according to those hrHPV types included in the Cervarix and Gardasil vaccines and those included in the Gardasil 9 vaccine.

### Data analysis

The mean age (± standard deviation), median and range, and other proportions including proportions for other baseline characteristics such as HIV status, parity and histologic subtypes were first computed. We examined the relationships of HIV status (positive or negative), age group and parity with hrHPV for all cervical cancers and by histologic subtype. Age was categorized as 30–40, 41–60, and ≥ 61 years. Parity was categorized as 0–5 and ≥ 6. Baseline characteristics were tested for association with hrHPV positivity using Fisher’s exact test.

A multivariable logistic regression model was used to calculate odds ratios (OR) and 95% confidence interval (95%CI) to measure the association of hrHPV positivity. P values of < 0.05 were considered statistically significant. Analyses were conducted using STATA 17 (StataCorp LLC, College Station, Texas, USA).

### Ethical considerations

This study protocol was submitted to the Muhimbili University of Science and Allied Sciences (MUHAS) institutional review board for review and approval (MUHAS-REC-11-2022-1432) and a permission to collect data was sought from the MNH management. A waiver of informed consent was requested and granted since the study involved archived FFPE tissue blocks and medical records without any contact with study participants. However, to ensure the confidentiality of all the patient information collected for study purposes, we used research codes for all data collected without any identifiers and kept the Excel sheet with the data in a password-protected computer with a document password as additional security. In addition, we applied for a material transfer agreement (MTA) from the National Institute of Medical Research (NIMR) **w**ith approval number: NIMR/HQ/R.8a/Vol.IX/4173 to transfer tissue sections to Rwanda for HPV genotyping.

## Results

**Fig. 1:**
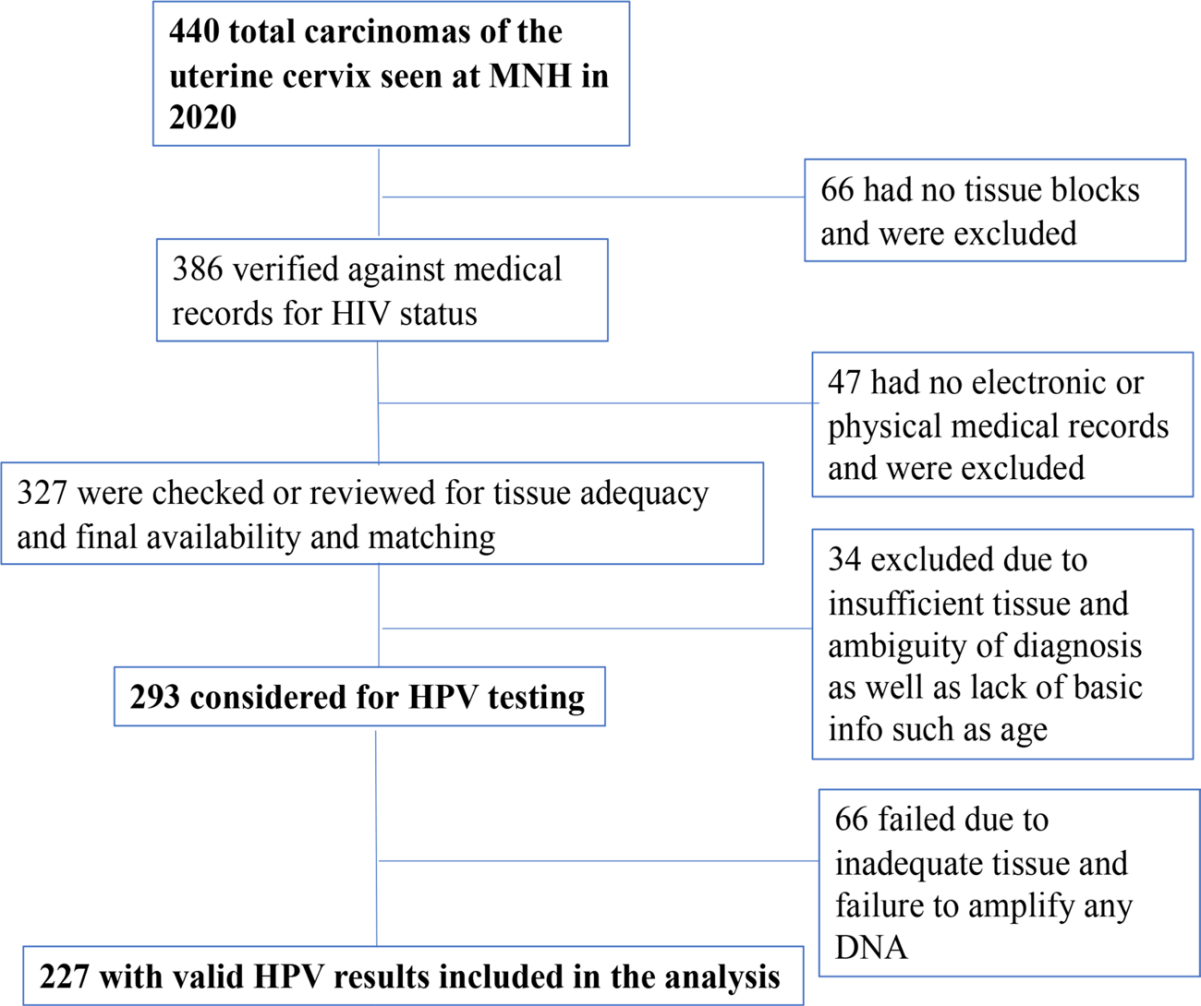
Flowchart for case selection

Records on a total of 440 cases of invasive cervical carcinoma seen at MNH from January to December 2020 were reviewed. Those cases were assessed for availability of tissue blocks, tissue adequacy, accuracy of diagnosis, HIV status, and other basic variables such as age according to the study inclusion and exclusion criteria. HPV DNA testing for 15 HPV types was performed on 293 cases that met the inclusion criteria. There were 227 (77.5%) valid HPV results, for the 14 hrHPV types excluding HPV53, which were included in the analysis (Fig. 1). No differences were seen for age, parity, and histologic subtypes between valid and invalid HPV results but more WLWHIV had invalid HPV results compared to HIV-negative women (43.0 vs. 10.0%, respectively, *p* < 0.001).

The mean age (*N* = 227) was 55 (± 12.9) years, with approximately half of the participants (53.3%) aged 41–60 years old. Most women (91 of 163, 55.8%) had given birth six or more times. Age (*p* = 0.04) and histologic subtype (*p* = 0.001) were positively associated with hrHPV positivity. Further details of the baseline characteristics of the study population by hrHPV positivity are presented in Table 1.

**Table 1:** Baseline characteristics of the study population, overall and by hrHPV positivity in cervical cancer cases from Muhimbili National Hospital in Tanzania

Characteristic/Categories	All: *N* = 227 *n* (col%)	hrHPV Negative *n* (row%)	hrHPV Positive *n* (row%)	*P* value*
**All**				
**Age groups (years)**				
30–40	31 (13.7)	11 (35.5)	29 (64.5)	**0.04**
41–60	121 (53.3)	18 (14.9)	103 (85.1)	
≥ 61	75 (33.0)	14 (18.7)	61 (81.3)	
**Parity**				
0–5	72 (31.7)	15 (20.8)	57 (79.2)	0.75
≥ 6	91 (40.1)	15 (16.5)	76 (83.5)	
Missing	64 (28.2)	13 (20.3)	51 (79.7)	
**HIV status**				
Negative	45 (19.8)	12 (26.7)	33 (73.3)	0.24
Positive	65 (28.6)	13 (20.0)	52 (80.0)	
Unknown	117 (51.5)	18 (15.4)	99 (84.6)	
**Histologic subtype**				
SCC	207 (91.2)	33 (15.9)	174 (84.1)	**0.001**
ADC†	20 (8.8)	10 (50.0)	10 (50.0)	

*Abbreviations* hrHPV, high-risk human papillomavirus; HIV, human immunodeficiency virus; SCC, squamous cell carcinoma; ADC, adenocarcinoma

*Fisher’s exact test comparing hrHPV negative vs. positive

†Includes 4 cases of adenosquamous carcinoma

Among the 227 with valid hrHPV results, 13 of the 14 hrHPV types were detected and the overall hrHPV positivity in cervical carcinomas diagnosed at MNH in 2020 was 81.1% (*n* = 184). The most common HPV types detected were HPV16 (*n* = 100, 44.1%), HPV18 (*n* = 36, 15.9%), HPV35 (*n* = 19, 8.4%), and HPV45 (*n* = 13, 5.7%). The other hrHPV types had proportions < 5%. Table 2 presents details of proportions for overall and individual hrHPV types. Supplementary Table 1 shows the most common hrHPV types (16, 18, 35 and 45) detected and their relationship with other hrHPV types.

**Table 2:** Distribution of high-risk HPV types by HIV status in cervical cancer cases from Muhimbili National Hospital in Tanzania

hrHPV type detected	All {*n* = 227, 100%} *n* (col%)	HIV negative {*n* = 45; 19.8%} *n* (col%)	HIV positive {*n* = 65, 28.6%} *n* (col%)	HIV unknown {*n* = 117, 51.6%} *n* (col%)	*P* value*
Any hrHPV	184 (81.1)	33 (73.3)	52 (80.0)	99 (84.6)	0.49
HPV16	100 (44.1)	14 (31.1)	28 (43.1)	58 (49.6)	0.24
HPV18	36 (15.9)	8 (17.8)	11 (16.9)	17 (14.5)	1.00
HPV31	0	0	0	0	n/a
HPV33	5 (2.2)	2 (4.4)	1 (1.5)	2 (1.7)	0.57
HPV35	19 (8.4)	2 (4.4)	6 (9.2)	11 (9.4)	0.47
HPV39	3 (1.3)	2 (4.4)	0 (0)	1 (0.9)	0.17
HPV45	13 (5.7)	4 (8.9)	1 (1.5)	8 (6.8)	0.16
HPV51	1 (0.4)	0 (0)	0 (0)	1 (0.9)	n/a
HPV52	4 (1.8)	1 (2.2)	2 (3.1)	1 (0.9)	1.00
HPV56	1 (0.4)	0 (0)	0 (0)	1 (0.9)	n/a
HPV58	10 (4.4)	3 (6.7)	6 (9.2)	1 (0.9)	0.74
HPV59	2 (0.9)	0(0)	1 (1.5)	1 (0.9)	1.00
HPV66	3 (1.3)	0(0)	1 (1.5)	2 (1.7)	1.00
HPV68	10 (4.4)	0(0)	7 (10.8)	3 (2.6)	**0.04**
**Number of hrHPV types detected**					
1	162 (88.0)	30 (90.9)	40 (76.9)	92 (92.9)	0.15
2 or more	22 (12.0)	3 (9.1)	12 (23.1)	7 (7.1)	

*Abbreviations* hrHPV, high-risk human papillomavirus; HIV, human immunodeficiency virus; n/a, not available

*Fisher’s exact test comparing HIV negative vs. positive

For all cases with valid HPV results (*N* = 227), HIV status was verified through both electronic and paper medical records: 19.8% (*n* = 45) were HIV negative, 28.6% (*n* = 65) were HIV positive, and 55.1% (*n* = 117) were of unknown HIV status. Table 2 shows the results stratified by HIV status. Among the 110 cases with known HIV status, hrHPV was detected in 85 (77.3%). HPV68 positivity was associated with HIV positivity (*p* = 0.04). Cases from WLWHIV were non-significantly more likely to test positive for two or more hrHPV types than HIV-negative women (*p* = 0.15).

Among the 227 women with valid HPV results, 91.2% (*n* = 207) had squamous cell carcinomas (SCC), 7.0% (*n* = 16) had adenocarcinomas (ADC), and 1.8% (*n* = 4) had adeno-squamous carcinomas (ADS) (Table 3). For simplicity and improving the power to make comparisons, ADC and ADS were combined in one group. SCC was more likely than ADC/ADS to test positive for hrHPV (84.1% vs. 50.0%, respectively, *p* = 0.001) and HPV16 (47.8% vs. 5.0%, respectively, *p* < 0.001).

**Table 3:** Distribution of HPV types by histologic subtypes in cervical cancer cases from Muhimbili National Hospital in Tanzania

hrHPV type detected	ADC/ADS {*n* = 20; 8.8%} *n* (col%)	SCC: {*n* = 207; 91.2%} *n* (col%)	*P* value*
Any hrHPV	10 (50.0)	174 (84.1)	**0.001**
HPV16	1 (5.0)	99 (47.8)	**< 0.0001**
HPV18	5 (25.0)	31 (15.0)	0.33
HPV31	0	0	n/a
HPV33	0 (0)	5 (2.4)	1.00
HPV35	0 (0)	19 (9.2)	0.39
HPV39	0 (0)	3 (1.5)	1.00
HPV45	3 (15.0)	10 (4.8)	0.1
HPV51	0 (0)	1 (0.5)	1.00
HPV52	0 (0)	4 (1.9)	1.00
HPV56	0 (0)	1 (0.5)	1.00
HPV58	1 (5.0)	9 (4.4)	1.00
HPV59	0 (0)	2 (1.0)	1.00
HPV66	0 (0)	3 (1.5)	1.00
HPV68	0 (0)	10 (4.8)	0.61
**Number of hrHPV types detected**			
1	10 (100)	152 (87.4)	0.61
2 or more	0 (0)	22 (12.6)	

*Fisher’s exact test; SSC: Squamous Cell Carcinoma; ADC: Adenocarcinoma; ADS: Adeno-squamous carcinoma

We also compared hrHPV types in currently available HPV vaccines (both first-and-second generation vaccines) and hrHPV types ordered hierarchically according to carcinogenicity, overall and stratified by HIV status and histologic subtype (Table 4). We found no statistically significant difference by HIV status and types included in first- and second-generation HPV vaccine by histologic subtype, but there was an association between hierarchical types and histologic subtype (*p* = 0.001).

**Table 4:** Categories of hrHPV by HIV status and histologic subtype among cervical cancer cases that tested hrHPV positive

hrHPV category	All: {*n* = 184, 100%} *n* (col%)	†HIV- {*n* = 33, 17.9%} *n* (col%)	†HIV+ {*n* = 52, 28.3%} *n* (col%)	HIV unknown {*n* = 99, 53.8%} *n* (col%)	*P* value	ADC/ADS {*n* = 10, 5.4%} *n* (col%)	SCC {*n* = 174; 94.6%} *n* (col%)	*P* value*
**1st Generation HPV Vaccines**								
HPV16/18	133 (72.3)	22 (66.7)	37 (71.1)	74 (74.7)	0.63	6 (60)	127 (73)	0.47
Non-HPV16/18	51 (27.7)	11 (33.3)	15 (28.9)	25 (25.3)		4 (40)	47 (27)	
**2nd Generation HPV Vaccines**								
HPV16/18/31/33/45/52/58	160 (87)	30 (90.9)	45 (86.5)	85 (85.9)	0.80	10 (100)	150 (86.2)	0.36
Non-HPV16/18/31/33/45/52/58	24 (13)	3 (9.1)	7 (13.5)	14 (14.1)		0 (0)	24 (13.8)	
Hierarchical**					0.39			
HPV16	100 (54.3)	14 (42.4)	28 (53.9)	58 (58.6)		1 (10)	99 (56.9)	**0.001**
HPV18/45	45 (24.5)	12 (36.4)	10 (19.2)	23 (23.2)		8 (80)	37 (21.3)	
HPV31/33/35/52/58	29 (15.8)	6 (18.2)	9 (17.3)	14 (14.1)		1 (10)	28 (16.1)	
Other hrHPV	10 (5.4)	1 (3.0)	5 (9.6)	4 (4.1)		0 (0)	10 (5.7)	

*Fisher’s exact test

**HPV16 positive, else HPV16 negative but HPV18 or HPV45 positive, else HPV16, 18, and HPV45 negative but HPV31, 33, 35, 52, or HPV58 positive, else negative for HPV16, 18, 31, 33, 35, 45, 52, and 58 but positive for other hrHPV types

†HIV status in which unknown was combined with negative and sensitivity analysis showed no differences when only known HIV status used; SSC: Squamous Cell Carcinoma; ADC: Adenocarcinoma; ADS: Adeno-squamous carcinoma

A multivariable logistic regression analysis was performed for variables associated with hrHPV positivity (age and histologic subtype) (Table 5). Cases from women who were aged 41–60 years (OR = 3.4, 95%CI = 1.35–8.40) and 61 years and older (OR = 3.0, 95%CI = 1.12–8.01) were more likely to test hrHPV positive than women aged < 40 years. SCC cases were more likely to test hrHPV positive than ADC/ADS cases (OR = 5.8, 95%CI = 2.17–15.49).

**Table 5:** Multivariable logistic regression analysis of variables associated with hrHPV positivity in cervical cancer cases from Muhimbili National Hospital in Tanzania

Characteristic/categories	Crude odds ratio (95% CI)	*P* value	Adjusted odds ratio (95% CI)	*P* value
**Age Group (Years)**				
30–40	Ref.	-	Ref.	-
41–60	3.2 (1.29;7.66)	0.012	3.4 (1.35;8.4)	**0.009**
≥ 61	2.4 (0.94;6.12)	0.068	3.0 (1.12;8.01)	**0.029**
**Histologic Subtype**				
ADC/ADS	Ref.	-	Ref.	-
SCC	5.3 (203;13.66)	0.001	5.8 (2.17;15.49)	**< 0.0001**

SSC: Squamous Cell Carcinoma; ADC: Adenocarcinoma; ADS: Adenosquamous carcinoma; 95% CI: 95% confidence interval; Ref.: Reference group

## Discussion

In the current study, we performed a molecular analysis to assess the proportions of overall and type-specific hrHPV positivity in cervical carcinomas diagnosed at MNH in 2020 and their differences by HIV status and histologic subtypes. The study showed a high proportion of cervical carcinomas positive for hrHPV (81.1%), greater hrHPV positivity with older ages, and greater hrHPV positivity in SCC than ADC/ADS.

These findings add to the limited data on hrHPV positivity in cervical cancer tissue in Tanzania and in sub-Saharan Africa (SSA). Prior evidence indicates that all cervical cancer (≥ 99%) was caused by hrHPV infection [9]. However, the recent WHO classification of female genital tumors recommends that all SCC and ADC, as well as ADC precancerous lesions (adenocarcinoma in situ) be classified into HPV-associated and HPV-independent categories. This can be done using p16 immunohistochemistry (as a surrogate marker for HPV) or HPV testing using PCR. The rationale underlying this recommendation is that certain carcinomas of the uterine cervix may not be associated with HPV as previously thought and that these carcinomas may have different biologic behavior, prognosis and perhaps therapeutic modalities [26] hence the need to gather information on them to be able to further characterize them.

First and foremost, baseline characteristics of this study population showed that hrHPV positivity was higher among cancers occurring among older women, and this is different from cervicovaginal HPV infection (using swabs) which has been shown to be higher among younger women and decreased with increasing age [28–30]. However, this finding is different from a large study done on cervical cancer tissue which showed that cancers due to HPV16/18/45 were more likely to occur at a younger age [31]. This unusual association between age and hrHPV positivity could not be explained in the context of existing evidence and it should be treated with caution as more studies and evidence emerge.

Histologic subtype was also associated with hrHPV positivity with SCC more likely to be associated with hrHPV; this is consistent with current evidence which suggests that adenocarcinomas are more diverse and some of them may be from the lower uterine segment and not entirely endocervical adenocarcinomas [26] or are rare HPV-negative cervical cancers that have endometrial cancer-like features [32]. Notably, 10 of the 20 ADC/ADS tested hrHPV negative, consistent with the latter explanation.

This study found that 81.1% of the cervical cancers seen at MNH in 2020 were positive for hrHPV. This finding is different from other studies which found hrHPV positivity over 90% [9, 15, 33] but some other studies, e.g. a large study by de Sanjose et al., found a similar proportion (85%) [34]. The most common hrHPV type was HPV16 followed by HPV18, 35 and 45 and these findings are similar to a study done in similar settings (Africa) [14] but different from a study published in 1992 and done in Tanzania, which showed almost equal proportions of HPV16 and 18 [35]. It also differs from other studies in other settings which found, in order of decreasing frequency, HPV16, 18, 31, 33, 35, and 45 [9, 15, 34, 36]. This suggests that the distribution of hrHPV types might differ in the SSA setting. However, the lower HPV16 and 18 positivity could be due to slightly higher proportions of other types such as HPV35 and 45 which are more common in African populations ( [17–19, 37]. Previous studies found that HPV16 and 18 cause up to 70% of all cervical cancers [31, 34] but in our case series we found that only 60% were due to HPV16 and 18, 44% due to HPV16. These proportions are slightly lower than global percentages for HPV16 and HPV18, which range from 70 to 80% [33, 34, 38]. Certain studies have even found HPV16 alone to cause up to 73% of all cervical cancers [38] indicating that our proportions are lower than those found in other settings.

Overall and type-specific hrHPV positivity by HIV status showed no differences except for HPV68 which appears to be more common in cases from WLWHIV than those from HIV-negative women. These findings are consistent with a study done in Rwanda on cervical cancer tissue [14]. These findings, however, are also different from other studies which found associations between HIV positivity and hrHPV positivity when using cervico-vaginal swabs and not cervical cancer tissue [28, 29].

Finally, the finding of more than 90% SCC with the rest being ADC and ADS is consistent with findings from other studies [15]. Furthermore, the associations found between histologic subtype and overall hrHPV and HPV16 positivity, with SCC more likely to be hrHPV and HPV16 positive, are somewhat different from findings from other studies in which ADC was found to be more likely to harbor HPV compared to all cervical carcinomas put together [31, 36]. In addition, evidence suggests that most ADC harbor more HPV18 than HPV16 [9, 26, 39]. We also found more HPV18 in ADC/ADS than SCC although the difference was not statistically significant; restricting to hrHPV-positive cases, HPV18 was more common in ADC/ADS than SCC (*p* < 0.001).

This study has some limitations which warrant mention. First, we could test slightly over half of the 440 cervical carcinomas recorded at MNH in 2020, which may imply that our findings may perhaps be different if all the other cases were to be included. In addition, it is important to note that the sensitivity of HPV testing using FFPE tissue is lower compared to testing on cervical-vaginal swabs which may partly explain the lower proportions of HPV positivity in our study [34]. Of note, tissue specimens from WLWHIV had more failed HPV tests compared to HIV-negative women which has the potential of biasing our findings, which we cannot explain but may suggest that cases from WLWHIV were less well handled and fixed. Second, we had over 50% women for whom we could not ascertain HIV status after verification of all available records.

Nonetheless, this study is among the first to study hrHPV positivity and types in cervical cancer tissue in Tanzania and perhaps in SSA with only two studies (found in recent literature within the past three decades), one done in South Africa [15] comparing two HPV molecular testing modalities on cervical cancer tissue and another one in Rwanda [14] comparing various anogenital anatomical sites.

Therefore, findings from this study are an important contribution to the scientific body of knowledge on the topic when a single dose of the HPV vaccine is being proved globally, and recommended by the WHO, to offer sufficient protection against HPV infection [40, 41]. With the cost and logistical challenges of providing more than one HPV vaccine dose minimized by a single dose, perhaps it is time that SSA national governments consider introducing the nonavalent HPV vaccine which includes hrHPV types in addition to HPV16 and HPV18 to offer maximum protection to their populations. However, the concern that the nonavalent vaccine does not include HPV35 remains and further considerations to include it are paramount to optimize cervical cancer prevention and control, especially among women of African descent. This, of course, should be considered in the context of the possibility of cross-protection for HPV35 hence the need for further studies. Hopefully, the recent announcement by Merck [42] to conduct clinical trials of a novel investigational multi-valent HPV vaccine may put an end to that concern.

## Conclusions

We found a high proportion of hrHPV in cervical carcinomas diagnosed in Tanzanian women, albeit lower than global rates. HPV16 and 18, the most important HPV types for cervical cancer etiology, together accounted for the majority of the carcinomas but with positivity lower than global reports. Our findings suggest that efforts should be made either through GAVI or other promising options [43] to allow access to the nonavalent HPV vaccine, which includes five more hrHPV types and that covers over 90% of the risk to bridge some of the gap of the 25% risk found in our study that is not covered by the currently available 1st generation HPV vaccines in SSA.

### Electronic supplementary material

Below is the link to the electronic supplementary material.

Supplementary Material 1

## Data Availability

No datasets were generated or analysed during the current study.

## Abbreviations

ADC: Adenocarcinoma
ADS: Adeno-squamous carcinoma
AIDS: Acquired Immunodeficiency Syndrome
CDC: Centers for Disease Control and Prevention
CIN: Cervical Intraepithelial Neoplasia
CPL: Central Pathology Laboratory
DNA: Deoxyribo Nucleic Acid
HIV: Human Immunodeficiency Virus
HPV: Human Papillomavirus
hrHPV: High-Risk Human Papillomavirus
IARC: International Agency for Research on Cancer
ICC: Invasive Cervical Cancer
LMIC: Low-and-Middle-Income Countries
MNH: Muhimbili National Hospital
MUHAS: Muhimbili University of Health and Allied Sciences
NIMR: National Institute of Medical Research
PCR: Polymerase Chain Reaction
SCC: Squamous Cell Carcinoma
SSA: sub-Saharan Africa
USA: United States of America
WHO: World Health Organization
WLWHIV: Women living with Human Immunodeficiency Virus
