# Supplementary material for: High-risk human papillomavirus genotyping in cervical cancers in Tanzania
Source: Infect Agent Cancer. 2024 Aug 5;19:35. doi: 10.1186/s13027-024-00596-1 (PMC11301851; doi:10.1186/s13027-024-00596-1)
Supplement: Supplementary file 1 — Supplementary Material 1 [file 13027_2024_596_MOESM1_ESM.docx]

**Supplementary Table 1:** The four most common hrHPV types found and co-infection with other types in cervical cancer cases from Muhimbili University in Tanzania

|  | **HPV16 Positive** | | **HPV18 Positive** | | **HPV35 Positive** | | **HPV45 Positive** | |
| --- | --- | --- | --- | --- | --- | --- | --- | --- |
| **HPV types and multiple infections** | **N** | **%** | **N** | **%** | **N** | **%** | **N** | **%** |
| HPV16 | 83 | 83 |  |  |  |  |  |  |
| HPV16/18 | 3 | 3 |  |  |  |  |  |  |
| HPV16/35 | 4 | 4 |  |  |  |  |  |  |
| HPV16/45 | 1 | 1 |  |  |  |  |  |  |
| HPV16/Others | 9 | 9 |  |  |  |  |  |  |
| HPV18 |  |  | 30 | 91 |  |  |  |  |
| HPV18/35 |  |  | 1 | 3 |  |  |  |  |
| HPV18/Others |  |  | 2 | 6 |  |  |  |  |
| HPV35 |  |  |  |  | 14 | 100 |  |  |
| HPV45 |  |  |  |  |  |  | 11 | 92 |
| HPV45/Others |  |  |  |  |  |  | 1 | 8 |

*Disclaimer: Opinions expressed by the authors are their own and this material should not be interpreted as representing the official viewpoint of the U.S. Department of Health and Human Services, the National Institutes of Health, or the National Cancer Institute.*
